# The challenge of exercise (non-)adherence: a scoping review of methods and techniques applied to improve adherence to physical activity and exercise in people with inflammatory arthritis

**DOI:** 10.1093/rap/rkac096

**Published:** 2023-01-24

**Authors:** Hema Chaplin, Mandeep Sekhon, Emma Godfrey

**Affiliations:** Department of Psychology, Institute of Psychiatry, Psychology and Neuroscience, King’s College London, London, UK; Population Health Research Institute, St George’s, University of London, London, UK; Department of Psychology, Institute of Psychiatry, Psychology and Neuroscience, King’s College London, London, UK; Department of Physiotherapy, Faculty of Life Sciences and Medicine, King’s College London, London, UK

**Keywords:** adherence, exercise, measurement, musculoskeletal, physical activity, scoping review

## Abstract

**Objectives:**

The aims were to explore the nature of methods/techniques applied to improve adherence to physical activity (PA) and exercise in people with inflammatory arthritis and to identify whether studies were theory based and/or used behaviour change techniques (BCTs).

**Methods:**

Searches were undertaken of English language articles within four databases: Embase, Medline, PsycINFO and Cochrane. Articles were included if they assessed adherence to a PA and/or exercise intervention. A narrative synthesis of the findings is reported.

**Results:**

Of 1909 studies screened, 18 studies met inclusion criteria. Adherence was most frequently included as a secondary outcome. Reporting of adherence measures was poor, in that 13 studies did not use a validated measure of adherence, with only three validated measures being identified. The majority of studies were not theory driven (*n* = 13), although the health belief model was the most used theoretical framework (*n* = 5). Only two studies mentioned both theory and BCTs. Four studies reported components that were mapped onto BCTs, with goal setting being the most prevalent.

**Conclusion:**

This scoping review found that adherence to PA and/or exercise interventions was rarely the focus of research, despite its importance in maintaining health in people with inflammatory arthritis. Where research has been conducted in this area, serious shortcomings were revealed, in that psychological theory, evidence-based BCTs derived from theory and valid adherence measures were not used to inform intervention design and target adherence, meaning that interventions were suboptimal. These results suggest that there is considerable room for improvement and that more high-quality research is required to investigate determinants of adherence and develop impactful interventions.

Key messages

- Adherence to physical activity and exercise interventions is rarely the focus of research, despite the importance of this to maintaining health in people with inflammatory arthritis.
- Most studies do not use psychological theory and evidence-based behaviour change techniques to inform intervention design.
- Reporting of intervention components is poor, and most studies do not use validated measures of adherence.

## Introduction

Physical activity (PA) and exercise (defined as planned, purposeful PA, designed to improve or maintain physical fitness) are key management strategies for people with inflammatory arthritis (including SpA, RA and PsA). People with inflammatory arthritis are advised to complete ≥150 min of moderate-intensity PA per week, with strengthening and flexibility exercises twice a week [1–3], but adherence to this guidance is often low [4–6]. The World Health Organization (WHO) defines adherence as ‘the extent to which a person’s behaviour corresponds with agreed recommendations from a healthcare provider’ [7]. However, this definition has been refined for exercise adherence by Frost *et al.* [8] as ‘the extent to which individuals undertake a prescribed behaviour accurately and at the agreed frequency, intensity and duration’. Adherence to PA and exercise can be difficult to measure, and much of the evidence base does not assess adherence as a primary outcome, but only as a secondary outcome [9].

There are particular barriers to participation in PA and exercise for people with inflammatory arthritis, and many spend the majority of their time engaged in sedentary behaviour, meaning that non-adherence is a significant challenge [10–12]. Patients’ perceptions of facilitators and barriers to PA and exercise need to be understood better, and interventions need to be more tailored to address individual determinants of this behaviour [13]. Recent research suggests barriers and facilitators in people with inflammatory arthritis are related to psychological status, social support, disease level and environmental factors [14]. However, a more in-depth understanding of adherence to PA and exercise is required, because there was poor adherence to this even among those who had high adherence to medication [15].

Several studies have been designed to address this problem using interventions such as exercise prescription, patient education and behavioural counselling. However, systematic reviews of interventions have revealed variable levels of success, with limited exploration of the methods/techniques used to assess adherence [16–19]. Furthermore, it is difficult to determine which aspects of these interventions were effective or how and why they might have worked, because most have not applied theory or tested fidelity [20]. Poor reporting of intervention design makes it difficult to draw conclusions about the effectiveness of theory or to assess whether the correct theory was chosen [21]. Few studies have described using evidence-based behaviour change techniques (BCTs) [22], and psychological theory has not been used to inform selection of behavioural change targets [23, 24].

Many interventions to increase PA and exercise in people with inflammatory arthritis have demonstrated limited application of psychological theory and/or poor reporting, making it difficult to draw conclusions about the best strategies to use [20]. A scoping review, defined as ‘a form of knowledge synthesis that addresses an exploratory research question aimed at mapping key concepts, types of evidence, and gaps in research related to a defined area or field by systematically searching, selecting and synthesizing existing knowledge’ [25], is therefore appropriate for inflammatory arthritis, because this can examine how research is conducted on a certain topic or field, identify key characteristics or factors related to a concept and analyse knowledge gaps [26].

The objectives of this scoping review were to explore the nature of techniques/methods applied to improve adherence to PA and/or exercise in people with inflammatory arthritis and to identify whether studies were theory based and/or used BCTs.

## Methods

The PRISMA Extension for Scoping Reviews (Preferred Reporting Items for Systematic Reviews and Meta-Analysis (PRISMA)-ScR) [27, 28] was followed and reported accordingly (see Supplementary Table S1, available at *Rheumatology Advances in Practice* online). A protocol for this scoping review has not been published because it was not eligible for registration on Prospero.

### Search strategy

Search terms included adapted MeSH, keyword and wild card terms located in the title or abstract that reflected disease and outcome (e.g. adherence/compliance to physical activity/exercise) taken from two previous systematic reviews on a similar topic of interest [19, 29] (see Supplementary Data S2, available at *Rheumatology Advances in Practice* online, for full search strategy). Studies were retrieved by searching electronic databases [MEDLINE, PsychINFO, EMBASE, and Cochrane Central Register of Controlled Trials (CENTRAL)]. Databases were searched from conception to 18 January 2022. All search results (titles and abstracts) were exported into Rayyan software to be stored during the screening process.

### Eligibility criteria

Articles were included if they assessed adherence to a PA and/or exercise intervention. A full list of inclusion and exclusion criteria for study inclusion is shown in Table 1 using the population, intervention, comparison, outcome(s) and study design framework.

**Table 1.** Eligibility criteria for considering studies for this review

Parameter	Inclusion criteria	Exclusion criteria
Population	Participants with inflammatory arthritis diagnosed according to established criteria were included (i.e. adults ≥18 years old with RA, PsA or axial SpA) English language	Other health conditions besides inflammatory arthritis Participants <18 years old Not English language
Intervention	All types of clinician-guided or self-directed behaviour change interventions (defined as coordinated sets of activities designed to change specified behaviour patterns [30], which may or may not include recognized behaviour change techniques) Treatment groups must have received intervention content that has the aim of changing participant behaviour (e.g. any effort by the health-care professional or researchers to change, or support change, of a behaviour); these might include, but were not limited to, goal-setting activities or behaviour monitoring Intervention descriptions must include a specific, measurable prescription of physical activity or exercise (i.e. a set of planned, structured and repetitive movements to be followed for the duration of the intervention)	No physical activity or exercise intervention clearly described
Comparison	Not applicable: studies with or without comparison groups included	
Outcomes	Self-reported measure of adherence to physical activity and/or exercise at the end of the intervention Outcomes could be reported as exercise diaries, questionnaires, levels of physical activity by any validated measure (e.g. monitoring device, i.e. step-count, accelerometer)	Other types of adherence, such as medication adherence Adherence not explicitly reported
Study design	Randomized controlled trials, quasi-experimental trials, prospective cohort studies, retrospective cohort analyses and before–after trials that reported baseline and follow-up measurements of adherence to physical activity and/or exercise or physical activity/exercise levels in at least two groups, including qualitative	Laboratory studies using animal models or cells Conference abstracts

### Data collection and analysis

#### Selection of studies

Primary screening was undertaken by the first coder (H.C.), with a random sample of 10% of studies cross-checked by a second coder (M.S.) at the screening stage, which resulted in a 0.90 kappa level of agreement (strong) between the two coders (3) because there were only four discrepancies. Raters discussed discrepancies and reached agreement on the final studies included for the review.

#### Data extraction and analysis

With the use of a study-specific data extraction table, information about each study (e.g. author, year of publication, country, study design), patient population, description of the intervention, details of adherence assessment (e.g. adherence measurement, validation and study outcome type) and involvement of theory or BCTs were extracted by the first coder (H.C.). A random sample of 10% of studies were extracted by a second coder (M.S.). Only published data have been extracted, with no further data requests or confirmation from study authors undertaken.

A narrative synthesis is presented to describe the methodology used to assess adherence to PA and/or exercise interventions with descriptive statistics [31, 32]. Depending on the studies meeting inclusion, a mapping of the theory used and BCTs was also undertaken [22]. Given that the required data concerned methodology and reporting, there was no differentiation in how the data from either qualitative and quantitative studies were dealt with. A quality assessment or critical appraisal was not conducted, because the aim of this scoping review was to examine how research is conducted on adherence to PA and/or exercise interventions, to identify key characteristics or factors related to adherence assessment and to analyse knowledge gaps [31].

## Results

### Study selection

**Figure 1.**
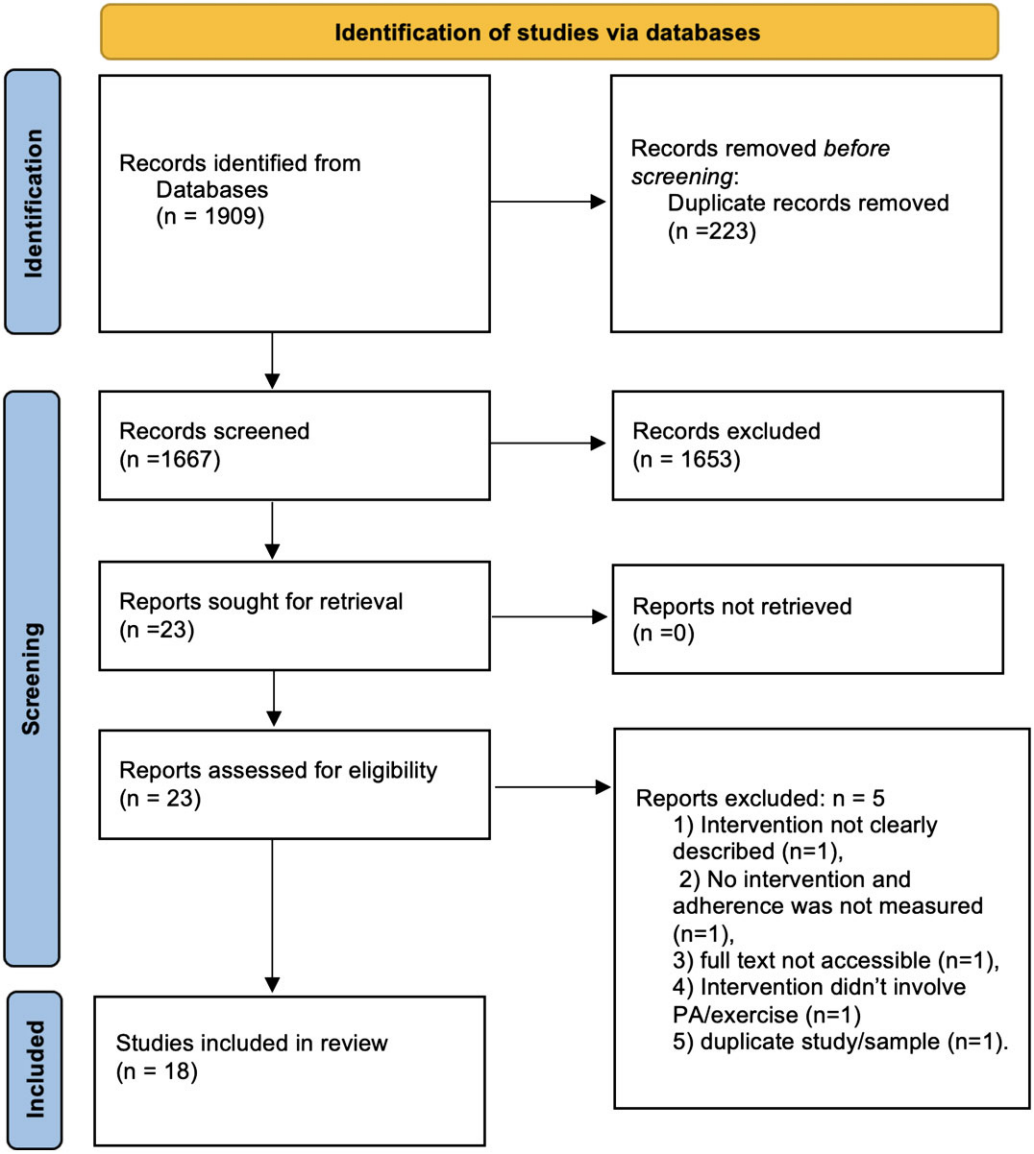
PRISMA flowchart

Combined searches yielded a total of 1909 citations, of which 1676 remained after removal of duplicates, with 23 studies meeting inclusion at the title/abstract screening stage (Fig. 1). At full-text screening and data extraction, 18 achieved final inclusion, with five studies being excluded for the following reasons: intervention not clearly described (*n* = 1); no intervention, and adherence was not measured (*n* = 1); full text not accessible (*n* = 1); intervention did not involve PA/exercise (*n* = 1); and duplicate study/sample (*n* = 1).

### Study characteristics

As shown in Table 2, the majority of included studies investigated RA (*n* = 12, 66.7%), mainly from Europe and UK (*n* = 13, 72.2%), with all studies published between 1999 and 2022. A variety of study designs were used, but the most common were randomized controlled trials or cross-over trials (*n* = 4, 22.2%). The sample size ranged from 14 to 328, with a median size of 42.5, with most studies (*n* = 14, 77.8%) representing a female majority of participants and with average age ranging from 21.54 to 63.6 years.

**Table 2.** Characteristics of included studies (*n* = 18)

Reference	Year	Country	Musculoskeletal population	Study type	Sample size	Age (years)	Female [*n* (%)]
[33]	2014	The Netherlands	Broad musculoskeletal (JIA, FM, RA, SpA and SLE)	Feasibility study	19	21.54 (range 17–25)	16 (84.2)
[34]^a^	2021	Norway and Sweden	SpA	RCT (secondary analysis)	100	46.2 (range 23–69)	53 (53)
[35]^a^	2020	Sweden	SpA	Qualitative analysis following RCT	14	53 (range 24–63)	5 (35.7)
[36]	2014	Italy	PsA	Observational cohort study	30	50.8 (s.d. 9.5)	12 (40)
[37]	2009	The Netherlands	RA	Observational study as long-term follow-up of RCT	71	56 (IQR 15)	61 (86)
[38]^a^	2004	UK	RA	Follow-up of cross-over trial	127	Not reported	97 (76.4)
[39]^a^	1999	UK	RA	Single-blind cross-over trial	35	55.17 (range 33–69)	29 (82.9)
[40]^a^	1999	UK	RA	Repeated measures, cohort design	21	48.95 (range 22–70)	17 (81)
[41]	2007	France	RA	RCT	208	54.7 (s.d. 13.1)	185 (88.9)
[42]	2017	USA	OA or RA	Qualitative analysis following pilot study	30	50 (range 32–69)	28 (93)
[43]	2001	Canada	RA	Pre–post experimental design	10	54 (s.d. 10)	10 (100)
[44]^a^	2017	UK	RA	Qualitative analysis following RCT	14	61.4 (range 44–82)	9 (64.3)
[45]	2013	Switzerland	SpA	RCT	106	48.85 (s.d. 12.16)	38 (35.8)
[46]	2015	Sweden	RA	Observational cohort study	220	59 (s.d. 8.8.)	178 (81)
[47]	2020	China	SpA	Cross-sectional study	259	33 (s.d. 17)	69 (26.6)
[48]	2022	USA	RA	Pilot RCT	50	56.1 (s.d. 11)	46 (92)
[49]	2017	New Zealand	RA	Assessor‐blinded, two‐arm pilot RCT	26	54 (range 29–73)	25 (96)
[50]^a^	2017	UK	RA	RCT, follow-up	328	63.6 (s.d. 10.9)	248 (75.6)

a Same research group/study but difference in samples and reporting.

IQR: interquartile range; RCT: randomized controlled trial; SpA: spondyloarthritis (axial or ankloysing).

### Synthesis of results from individual studies

Details of interventions, adherence assessments, theory and BCTs for each included study are presented in Table 3. A wide range of 14 interventions were described across the 18 studies, with most following physiotherapist- or occupational therapist-supervised moderate- to high-intensity exercise programmes (*n* = 5, 35.7%) or using predominantly education-based programmes (*n* = 4, 28.6%). Only one intervention was delivered exclusively online [32], with other interventions incorporating dance-based exercise [43] and Nordic walking [45] as PA and/or exercise.

**Table 3.** Details of interventions, adherence assessments, theory and behaviour change techniques from included studies (*n* = 19)

Reference	Intervention	Adherence measurement	Measure validated	Type of outcome	Associated behaviour change techniques	Theory involved
[33]	Online programme consisting of three e-Health applications, including a chat section, home exercises and a discussion board	Adherence to the programme was measured after completing the programmes by describing how many people had completed the whole course. Also, each participant’s presence during the chats on the discussion board and finishing the exercises of the online programme were measured (frequencies reported)	No	Secondary outcome	Goal setting (unspecified)	No
[34]^a^	Three-month physiotherapist-supervised high-intensity exercise programme	Exercise adherence was recorded by the physiotherapist as attendance at the supervised sessions and as accomplishment of the individual session of personal choice by inspection of the pulse watch. Exercise adherence was also self-reported by the participants in a personal exercise diary to enhance motivation. Reported as the percentage who followed ≥80% of the prescribed exercise protocol	No	Secondary outcome	No	Health beliefs model, because exercise health beliefs were the primary outcome
[35]^a^	Three-month physiotherapy supervised high-intensity exercise programme	Reported as the percentage who followed ≥80% of the prescribed exercise protocol	No	Secondary outcome	No	No
[36]	Exercise programme delivered by a single physiotherapist, with leaflets to facilitate correct performance of the exercises	Self-reported rates of adherence to a home-based programme of exercises (percentage)	No	Feasibility outcome	No	No
[37]	Two-year supervised high-intensity exercise programme	At 18 months of follow-up, all participants completed a 10-item questionnaire comprising questions on frequency, intensity and compliance with exercises, and the reasons for not continuing the participation in the RAPIT group and choice of an alternative if applicable. Patients reporting participation in extended RAPIT groups or other classes were asked to give the name of their supervisor, and their participation was checked with the lists of participants available from the providers	No	Primary outcome	No	No
[38]^a^	The JP group education programme consisted of four weekly 2-h sessions, plus an optional home visit within 2 weeks of the end of the programme. It was led by an experienced rheumatology occupational therapist covering RA, drug treatments, diet, exercise, pain management, relaxation and joint protection	Joint protection behaviour assessment: performances of 20 tasks when making a hot drink and snack meal were assessed as incorrect, partly correct or correct joint protection methods, with scores converted to percentages. A higher score indicates increased adherence	Yes	Primary outcome	No	Educational, behavioural, motor learning and self-efficacy enhancing strategies to increase adherence
[39]^a^	The JP group education programme consisted of four weekly 2-h sessions, plus an optional home visit within 2 weeks of the end of the programme. It was led by an experienced rheumatology occupational therapist covering RA, drug treatments, diet, exercise, pain management, relaxation and joint protection	Joint protection behaviour assessment: performances of 20 tasks when making a hot drink and snack meal were assessed as incorrect, partly correct or correct joint protection methods, with scores converted to percentages. A higher score indicates increased adherence	Yes	Primary outcome	Instruction on how to perform the behaviour, demonstration of the behaviour, feedback on behaviour, problem-solving, habit formation, goal setting (behaviour), behavioural contract and social support (unspecified), credible source (an experienced rheumatology therapist delivered intervention), information about health consequences, verbal persuasion about capability, behaviour practice/rehearsal, self-monitoring of behaviour	Group education programme was developed using the health belief model and self-efficacy theory
[40]^a^	The JP group education programme consisted of four weekly 2-h sessions, plus an optional home visit within 2 weeks of the end of the programme. It was led by an experienced rheumatology occupational therapist covering RA, drug treatments, diet, exercise, pain management, relaxation and joint protection	Joint protection behaviour assessment: performances of 20 tasks when making a hot drink and snack meal were assessed as incorrect, partly correct or correct joint protection methods, with scores converted to percentages. A higher score indicates increased adherence	Yes	Primary outcome	Instruction on how to perform the behaviour, demonstration of the behaviour, feedback on behaviour and problem-solving, behaviour practice/rehearsal, habit formation, information about health consequences	No
[41]	The active group received a multidisciplinary education programme, including training in home-based exercises and guidelines for leisure physical activity. The control group received a booklet added to usual medical care	Compliance with home-based exercises was defined as a practice rate ≥30% of the prescribed training. Compliance with leisure physical activity was defined as ≥20% increase in Baecke questionnaire score. Additional assessments involved possible predictors of compliance and changes with regard to the compliance Exercise compliance assessment at a given visit. The compliance rate for home-based exercise was measured as described [33]. The mean weekly practice was calculated as the proportion of self-reported mean weekly number of exercises to total number of exercises included in the home-based programme. To be compliant, each participant had to have a compliance rate ≥30%, meaning at least a daily mean practice of a set of three different exercises whatever the exercises performed and have disrupted training for <1 month before the 6-month follow-up visit and <2 months before the 12-month follow-up visit Leisure physical activity compliance was measured by comparing the baseline and follow-up (6- or 12-month) level of leisure physical activity as assessed by the Baecke questionnaire. Given that identification of a minimal clinically important difference is lacking for the Baecke score, we decided that compliant participants had to have increased their score by ≥20% over that at baseline. This threshold was chosen because of its clinical relevance and out of respect to the five-point scale of the Baecke questionnaire	No	Primary outcome	No	No
[42]	Eight-week group hatha yoga programme	Number completing intervention (not necessarily attending all sessions)	No	Feasibility outcome	No	No
[43]	The dance-based exercise programme was developed and led by a physical fitness instructor in collaboration with an occupational therapist and a physical therapist. Each training session included four phases, all taking place to musical arrangements: warm-up, aerobic exercise, recovery and cool-down. The dance-based exercise period was made up of slow movements, creating a rhythmic pattern that involved all joints	Compliance measured as rate of participation in sessions (descriptive)	No	Feasibility outcome	No	No
[44]^a^	Individually tailored moderate- to high-intensity strengthening and stretching exercises over five sessions with an occupational therapist or physiotherapist	Interview schedule questions: Was there anything that helped you to do the exercises regularly? Was there anything that made it difficult for you to do the exercises regularly? Did the exercise programme work for you? Why do you think that the exercise programme would work for some and not others? Themes/subthemes linked to adherence	No	Secondary outcome	Goal setting (unspecified) and behavioural contract	Educational behavioural model based on the health beliefs model
[45]	The training group performed a 12-week supervised Nordic walking training for 30 min twice a week using individually monitored, moderate-intensity heart rate levels	Based on the physiotherapists’ protocols for group adherence and on the participants’ diaries, the percentage who performed at least three training units per week (i.e. two Nordic walking training sessions and one additional unsupervised cardiovascular training unit)	No	Feasibility outcome	No	No
[46]	Three main components constituted the intervention programme: (i) at least moderate-intensity physical activity for ≥30 min on most days of the week; (ii) at least two weekly 45 min circuit training sessions, including both muscle strength training (50–80% of one repetition maximum, 3–10 repetitions) and aerobic exercises (60–85% of maximal heart rate); and (iii) biweekly support group meetings	Two text messages were sent once each week to collect data on the number of days during the past week that participants performed circuit training sessions and on how many additional days of the past week they performed at least moderate-intensity physical activity for ≥30 min. Support group meeting attendance was registered by the coaches. Participants were categorized into adherers and non-adherers based on 50, 70 and 90% participation in circuit training sessions, total HEPA and support group meetings, respectively	Yes (EMA)	Secondary outcome	No	No
[47]	Educated with the types of back exercise and the importance of adhering to standard exercise therapy by rheumatologists	Exercising for ≥30 min per day and performing back exercise on ≥5 days per week were defined as adherence to the standard exercise therapy	Yes	Secondary outcome	No	No
[48]	Twelve-session group programme covering pain coping skills, lifestyle behavioural weight loss plus supervised exercise sessions three times per week	Descriptive statistics (percentage)	No	Feasibility outcome	No	No
[49]	An 8‐week programme of group and home yoga practice. Group practice consisted of once‐weekly 75‐min yoga classes, conducted by a qualified yoga instructor and class assistant. Home practice consisted of a 20‐min guided relaxation, based on the relaxation technique practised in the group sessions. A CD, recorded by the yoga instructor, was provided. Participants were asked to practise three times per week, at a time and day of their choice	Protocol adherence (*a priori* level of 6/8 group classes and 16/24 home classes acceptable). Adherence to home practice in the previous week was reported verbally to the yoga instructor at the beginning of each session, and barriers and adherers to home practice were discussed among the group	No	Feasibility outcome	No	No
[50]^a^	Individually tailored moderate- to high-intensity strengthening and stretching exercises over five sessions with an occupational therapist or physiotherapist	To assess adherence to the exercise programme, all participants were asked to report how often they performed hand exercises for their RA (frequency, percentage)	No	Secondary outcome	No	Educational behavioural model based on the health beliefs model

a Same research group/study but difference in samples and reporting.

Adherence was most frequently included as a secondary outcome (*n* = 7, 38.9%), with only five studies reporting it as their primary outcome (27.8%). Most studies used the term adherence, with three studies using the term compliance (16.6%). Reporting of adherence measures was poor, with most studies not using a validated measure of adherence (*n* = 13, 72.2%), typically using a study-specific measure (*n* = 9, 69.2%) or simply presenting a descriptive statistic (usually a percentage or frequency) of those completing the intervention/course (*n* = 4, 30.8%), often only in the Discussion. The validated measures of adherence were as follows: joint protection behaviour assessment [38–40]; using ecological momentary assessment to capture frequency data alongside group attendance [46]; and a definition of adherence to standard exercise therapy stated as exercising for ≥30 min per day and performing back exercise on ≥5 days per week based on previous literature [47].

The majority of studies were not theory driven (*n* = 13, 72.2%); however, of those five that mentioned theory, all used the health belief model as the theoretical framework, either explicitly mentioned, alluded to or in conjunction with self-efficacy theory. Only two studies mentioned both theory and BCTs (10.5%) [39, 44]. Four studies in total (21%) mentioned components that can be mapped onto BCTs [33, 39, 40, 44], although they did not use the terminology of BCTs, ranging from 1 to 13 BCTs (median = 4.5) across studies, with goal setting the most common (*n* = 3, 75%).

It is important to note that three studies/interventions were exemplified across seven papers [34, 35, 38–40, 44, 50], with some papers representing follow-up (both qualitatively and quantitatively). This is particularly significant given that Hammond *et al.* [38–40] assessed adherence in a more theory-driven and rigorous way, with the use of BCTs to inform their intervention, compared with the rest of the literature. However, the level of detail in reporting was inconsistent across publications for the same/similar studies, particularly regarding theory usage, reporting of BCTs and interventions.

## Discussion

### Summary of evidence

Eighteen studies met inclusion criteria and had data extracted and analysed as part of this scoping review. A narrative synthesis was completed to describe the methodology used to assess adherence to PA and/or exercise interventions using descriptive statistics. Interestingly, adherence was reported as a primary outcome in only five studies and was most frequently included as a secondary outcome. Although the term adherence was used most commonly, reporting of adherence measures was poor, with most studies not using validated measures of adherence. In addition, many studies did not underpin interventions with theory, and the five that did all used the health belief model, sometimes in combination with self-efficacy theory. Four studies mentioned components that could be mapped onto BCTs [33, 39, 40, 44], although they did not always apply proper BCT terminology, ranging from 1 to 13 BCTs across studies. Goal setting was the most commonly used BCT, but only one paper [39] specified that goal-setting behaviour was used, whereas two others [33, 44] did not specify whether goal-setting behaviour or outcome was used. However, there was inconsistent reporting of this across publications, even within the same or similar studies, particularly regarding theory usage and reporting of BCTs and intervention components.

The scoping review reported similar shortcomings to Fenton *et al.* [20], because limited application of psychological theory and/or poor reporting made it difficult to draw conclusions about the best strategies to use to increase adherence to PA and exercise in people with inflammatory arthritis. However, this scoping review has added to the existing literature and advanced understanding by finding that psychological theory and evidence-based BCTs derived from theory have not been engaged to inform intervention design and target adherence. Furthermore, the included research did not distinguish between the initiation and maintenance of PA or exercise, which might be influenced by different determinants, and importantly, most studies did not use a validated measure of adherence.

### Implications of the scoping review

There are implications of this review for both researchers and clinicians. It is clear that researchers need to design interventions that are theory based, then to identify the specific BCTs impacting on PA- or exercise-adherent behaviour in order to change this behaviour, if they are to be effective [51]. There is also potential to improve future studies by using valid and reliable measures of adherence as primary or secondary outcomes; for example, the exercise adherence rating scale (EARS) [24, 52]. This measure has been widely validated, used across different populations and translated into several languages [53–55]. Clinicians could be trained to use effective BCTs [56] and could also use brief measures, such as the EARS, to improve and assess the success of their treatment, because adherence to PA and exercise is an important issue for people with inflammatory arthritis [11, 15].

### Strengths and limitations

An important strength of this scoping review was that it followed the PRISMA Extension for Scoping Reviews (PRISMA-ScR) process [27, 28]. In addition, to check for accuracy, a random sample of 10% of studies was extracted by a second coder (M.S.). One of the limitations of this scoping review is that no quality appraisal of studies was completed. However, this was not relevant to the aims of this scoping review, which was designed purely to examine how research was conducted on adherence to PA and/or exercise interventions, to identify key characteristics or factors related to adherence assessment and to analyse knowledge gaps [26]. It is also possible that some relevant research was not assessed, because only English language papers and published data were included, with no further data requests or confirmation from study authors undertaken. A further limitation is that a librarian or information specialist was not consulted when developing the search strategy, and therefore some key terms might have been missed, although this is unlikely given the authors’ experiential knowledge.

### Conclusions

This scoping review found that adherence to PA and/or exercise interventions was rarely the focus of research studies, despite the importance of PA and/or exercise to maintaining health in people with inflammatory arthritis. Where research has been conducted in this area, serious shortcomings were revealed, because in many studies psychological theory and evidence-based BCTs derived from theory were not used to inform intervention design and target adherence, meaning that interventions were suboptimal. Furthermore, reporting of intervention components and choice of adherence measures was poor, with most studies not using validated measures of adherence. These results suggest that there is considerable room for improvement in this area and that more high-quality research is required to investigate the determinants of PA and/or exercise adherence and develop targeted interventions to enhance it in people living with inflammatory arthritis. Researchers and clinicians should use valid and reliable measures and carry out theory-informed research that targets adherence accurately using appropriate BCTs, in order to improve the outcome and provide better support for people living with inflammatory arthritis.

## Supplementary Material

rkac096_Supplementary_Data

## Data Availability

The secondary data generated that support the findings of this study are available from the corresponding author upon reasonable request.
