# Supplementary material for: The challenge of exercise (non-)adherence: a scoping review of methods and techniques applied to improve adherence to physical activity and exercise in people with inflammatory arthritis
Source: Rheumatol Adv Pract. 2023 Jan 24;7(1):rkac096. doi: 10.1093/rap/rkac096 (PMC9880978; doi:10.1093/rap/rkac096)
Supplement: rkac096_Supplementary_Data [file rkac096_supplementary_data.zip › rkac096_Supplementary_Data/22-060 Supplementary Data S2.docx]

**Supplementary Data S2. Search strategy**

**MEDLINE, EMBASE, PsycARTICLES and PsycINFO:**

| 1. exp arthritis, rheumatoid/ |
| --- |
| 1. ((rheumatoid or reumatoid or revmatoid or rheumatic or reumatic or revmatic or rheumat$ or reumat$ or revmarthrit$) adj3 (arthrit$ or artrit$ or diseas$ or condition$ or nodule$)).tw. |
| 1. exp Spondylitis, Ankylosing/ |
| 1. axial spondylo*.mp. |
| 1. (ankylos$ or spondyl$).tw. |
| 1. Arthritis, Psoriatic/ |
| 1. (psoria$ adj2 (arthriti$ or arthropath$)).tw. |
| 1. Exp Arthritis, Juvenile/ |
| 1. (juvenile adj2 arthritis).tw. |
| 1. Arthritis, Inflammatory/ |
| 1. (Juvenile inflammatory Arthritis or JIA).mp. [mp=title, abstract, original title, name of substance word, subject heading word, floating sub-heading word, keyword heading word, organism supplementary concept word, protocol supplementary concept word, rare disease supplementary concept word, unique identifier, synonyms] |
| 1. or/ 1-11 |
| 1. exp Behavior Therapy/ or exp Cognitive Behavior Therapy/ or exp Cognitive Therapy/ |
| 1. "behavio?r therapy".mp. [mp=title, abstract, original title, name of substance word, subject heading word, keyword heading word, protocol supplementary concept word, rare disease supplementary concept word, unique identifier, synonyms] |
| 1. "behavio?r treatment".mp. [mp=title, abstract, original title, name of substance word, subject heading word, keyword heading word, protocol supplementary concept word, rare disease supplementary concept word, unique identifier, synonyms] |
| 1. "behavio?r taxonomy".mp. [mp=title, abstract, original title, name of substance word, subject heading word, keyword heading word, protocol supplementary concept word, rare disease supplementary concept word, unique identifier, synonyms] |
| 1. "cognitive treatment".mp. [mp=title, abstract, original title, name of substance word, subject heading word, keyword heading word, protocol supplementary concept word, rare disease supplementary concept word, unique identifier, synonyms] |
| 1. exp Self-Concept/ |
| 1. exp Self-Efficacy/ |
| 1. exp Goals/ or exp Goal Setting/ |
| 1. ("self monitor$" or "self regulat$" or "self talk").mp. [mp=title, abstract, original title, name of substance word, subject heading word, keyword heading word, protocol supplementary concept word, rare disease supplementary concept word, unique identifier, synonyms] |
| 1. exp EXERCISE/ |
| 1. exp Physical Activity/ |
| 1. "physical activ*".mp. [mp=title, abstract, original title, name of substance word, subject heading word, keyword heading word, protocol supplementary concept word, rare disease supplementary concept word, unique identifier, synonyms] |
| 1. adhere*.mp. [mp=title, abstract, original title, name of substance word, subject heading word, keyword heading word, protocol supplementary concept word, rare disease supplementary concept word, unique identifier, synonyms] |
| 1. ("non adherence" or "non-adherence").mp. [mp=title, abstract, original title, name of substance word, subject heading word, keyword heading word, protocol supplementary concept word, rare disease supplementary concept word, unique identifier, synonyms] |
| 1. ("concordance" or "cooperate" or "co-operate engage" or "disengage").mp. [mp=title, abstract, original title, name of substance word, subject heading word, keyword heading word, protocol supplementary concept word, rare disease supplementary concept word, unique identifier, synonyms] |
| 1. (attendance or appointment).mp. [mp=title, abstract, original title, name of substance word, subject heading word, keyword heading word, protocol supplementary concept word, rare disease supplementary concept word, unique identifier, synonyms] |
| 1. behavio?r change".mp. [mp=title, abstract, original title, name of substance word, subject heading word, keyword heading word, protocol supplementary concept word, rare disease supplementary concept word, unique identifier, synonyms] |
| 1. ("self-monitor*" or "self monitor*").mp. [mp=title, abstract, original title, name of substance word, subject heading word, keyword heading word, protocol supplementary concept word, rare disease supplementary concept word, unique identifier, synonyms] |
| 1. Exp compliance/ or exp Patient compliance |
| 1. 13 or 14 or 15 or 16 or 17 or 18 or 19 or 20 or 21 or 29 or 30 or 31 |
| 1. 22 or 23 or 24 or 25 or 26 or 27 or 28 |
| 1. 12 and 32 and 33 |
| 1. Limit 34 to English language |

**Cochrane Database**

| S1 MeSH descriptor Arthritis, explode all trees |
| --- |
| S2 ((rheumatoid or reumatoid or revmatoid or rheumatic or reumatic or revmatic or rheumat* or reumat* or revmarthrit*) W3 (arthrit* or artrit* or diseas* or condition* or nodule*)) |
| S3 MeSH descriptor, Spondyloarthropathies explode all trees |
| S4 arthritis or ankylosing nxt spindly* or rheumatic next disease* or rheumatic next disorder* or rheumatic next condition* |
| S5 #1 or #2 or #3 or #4 |
| S6 MeSH descriptor, Behavior Therapy explode all trees |
| S7 “behaviour change taxonomy” |
| S8 MeSH descriptor, Cognitive Therapy explode all trees |
| S9 MeSH descriptor, Self efficacy explode all trees |
| S10 MeSH descriptor, Self Concept, explode all trees |
| S11 “goal setting” |
| S12 “goal attainment” |
| S13 “MeSH descriptor, Exercise explode all trees |
| S14 “adhere” or “adherence” or “adhering” |
| S15 compliance |
| S16 concordance |
| S17 “cooperate” or “Co-operate” |
| S18 Engage |
| S19 attendance |
| S20 appointment |
| S21 “behaviour treatment” or “behavior treatment” ti, ab, kw (Word variations have been searched) |
| S22 psychological interventions ti, ab, kw (Word variations have been searched) |
| S23 “self talk” or “self monitoring” or “self-regulation” ti, ab, kw (Word variations have been searched) |
| S24 Physical activity ti, ab, kw (Word variations have been searched) |
| S25 {or #6-#12, #21-23} |
| S26 {or #13-#20, #24} |
| S27 {and #25-#26} |
| S28 {and #5, #27} |
